# What plays a role in the severity of atopic dermatitis in children?

**DOI:** 10.3906/sag-2101-194

**Published:** 2021-10-21

**Authors:** Murat CANSEVER, Çiğdem ORUÇ

**Affiliations:** 1 Division of Pediatric Allergy and Immunology, Department of Pediatrics, Mardin State Hospital, Mardin Turkey; 2 Department of Pediatrics, Mardin State Hospital, Mardin Turkey

**Keywords:** Atopic dermatitis, children, food allergy, risk factors

## Abstract

**Background/aim:**

Determining the characteristics and risk factors of severe disease is extremely important to combat atopic dermatitis (AD), which has recently shown increasing prevalence. In this study, we aimed to investigate the clinical characteristics of pediatric patients with AD and identifying the factors associated with the severity of the disease.

**Materials and methods:**

A total of 304 pediatric patients diagnosed with atopic dermatitis were included in the study. The patients’ age at admission, age at onset of symptoms, the presence of atopy history in their family, eosinophil levels obtained from blood counts were recorded, together with the data of cigarette exposure, and area of residence. Disease severity was determined according to the SCORAD index. Epidermal prick tests (EPT) were applied to all patients.

**Results:**

There was a negative correlation between the SCORAD score and both age at admission (r = 0.277, p < 0.001) and age at onset of the symptoms (r = –0.474, p < 0.001). Food sensitization rates were higher in individuals with moderate-severe disease (90.7% vs. 23.1%; p < 0.001) and patients with food allergy (FA) had significantly higher SCORAD scores [33 (IQR: 22–44) vs. 14 (IQR: 12–16); p < 0.001]. SCORAD scores of the individuals living in rural areas were higher than the ones living in urban [22 (IQR: 15–39.5) vs. 15 (IQR: 12–22); p < 0.001]. Familial atopy history was more common in patients with moderate-severe disease (66.5% vs. 17.5%; p < 0.001). The SCORAD scores were higher in patients exposed to passive smoking [21 (IQR:14.75–38) vs. 13 (IQR: 12–16); p < 0.001]. The eosinophil count found to be positively correlated with SCORAD scores (r = 0.531, p < 0.001).

**Conclusion:**

Our findings show that early-onset, food sensitivity, living in rural areas, having familial atopy history and passive cigarette smoke exposure play a role in severe AD. Since it is remarkably correlated with SCORAD scores, eosinophil count can be used as a marker to assess the severity of AD in children.

## 1. Introduction

Atopic dermatitis (AD) is a chronic relapsing, pruritic, inflammatory skin disease [1]. This disorder is associated with many comorbid conditions leading to impaired overall health and increased healthcare utilization [2]. It has been one of the most common chronic diseases of the present time and affects 15%–20% of the child population worldwide [3]. The onset is most commonly observed at the age of 3 to 6 months, with approximately 60% of patients developing eczema in the first year of their lives and 90% of patients by the age of 5 [1]. 

In order to prevent a disease that is remarkably common and has wide detrimental effects, it is essential to determine the risk factors of the disease, however, the pathogenesis of AD is not entirely understood [4]. 

With this single-centered cross-sectional study, we aimed at investigating the clinical characteristics of pediatric patients with AD and identifying the factors associated with the severity of the disease.

## 2. Materials and methods

### 2.1. Clinical assessment

In our retrospective cross-sectional study, the data of the patients who applied between December 2019 and December 2020 were obtained from the hospital records. A total of 304 pediatric patients attending to Mardin State Hospital Pediatric Allergy Outpatient Clinic between December 2019 and December 2020, and diagnosed with AD according to Hanifin Rajka criteria [5,6], at the time of admission were included in this study. The individuals with a previous diagnosis of AD, with dermographism, having dermatological diseases other than AD, under treatment, or having a special diet were excluded. 

The data on patients’ age at admission, age at onset of symptoms, the presence of atopy history in their family, the cigarette smoke exposure, and area of residence were collected from the patients’ records. Family history of atopy was defined as having at least one parent or sibling with physician-diagnosed asthma, AD and/or allergic rhinitis [7]. Disease severity was determined according to the SCORAD index: The patients with a score below 25 were classified as mild, 25–50 as moderate, and above 50 as severe AD [8].

The eosinophil levels and eosinophil percentages were obtained from the complete blood counts. Epidermal prick tests (EPT) were performed on all of the patients. The panels consisting of the 16 most common allergens in Turkey including food allergens (cow’s milk, egg white, egg yolk, wheat, hazelnut, peanut, soybean, fish species, sesame, walnut) and aeroallergens (*Dermatophagoides farinae*, *Dermatophagoides pteronyssunus*, *Alternaria tenuis*, *Penisilium notatum*, *Aspergillus fumigatus*) were used for EPT [9]. Histamine was used as positive control while 0.9% NaCl solution as negative control. The commercial extracts (Allergopharma; D-21462 Reinbek, Germany) were applied for EPT. The prick tests were performed to the volar or dorsal of the forearms after cleaning the areas with alcohol and by using skin prick test applicators (Medblue one 020013, Turkey). The wheal diameter that occurred 15 min after the tests had been applied was measured. The test was considered positive if the wheal diameter was at least 3 mm greater compared to the negative control [10]. Patients with positive results in EPT were accepted as “sensitized”. All epidermal prick tests were done and evaluated by the same clinician. Thus, standardization was achieved in the application and evaluation of the tests.

The individuals found to be “sensitive” in the epidermal prick tests were undergone oral food challenge (OFC) with the food allergen to which they were sensitive. Those who had positive results in OFC were diagnosed with a food allergy (FA).

The study protocol was approved by the Ethical Committee of Artuklu University, Turkey. Informed written consent was obtained from the caregivers of the participants.

### 2.2. Statistical analyses

Statistical analyses were performed using SPSS v. 20.0 (IBM Corp., Armonk, NY, USA). Results were indicated as means (standard deviations) for variables with a normal distribution and as medians (interquartile range) for variables with skewed distribution. The statistical significance of differences between the groups was tested using the Chi-squared test and Fisher’s exact test for categorical parameters; Mann–Whitney U test and Kruskal–Wallis test for continuous parameters. Spearman’s rank correlation coefficients were assessed as measures of correlation between variables of interest. To assess factors associated with SCORAD scores we applied linear regression modeling. The variables that reach a p-value < 0.10 in the univariate analysis were included in a multiple linear regression analysis to investigate factors independently associated with SCORAD scores. Values of p < 0.05 were considered to be statistically significant.

## 3. Results

A total of 304 pediatric patients with AD were enrolled in the study. The mean age at admission was 8.1 (± 4.8) months and the mean age at the onset of symptoms was 4.5 (± 3) months. There was a slight male predominance in the whole study group (64% male, 36% female). 

The median SCORAD score of the whole study group was 16 (IQR: 13–24). According to the classification of AD severity, 75.3% (229) of the patients had mild, 20.4% (62) had moderate, and 4.3% (13) had severe AD. 

**Figure 1:**
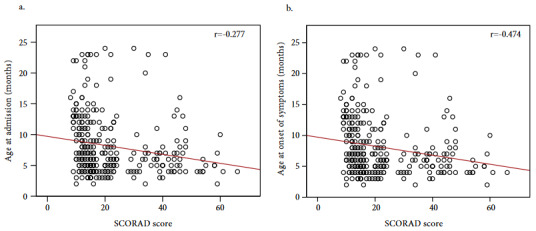
Correlation between the SCORAD score and age [(a) at admission and (b) at the onset of the symptoms] (r, Spearman’s rank correlation).

The ages at admission and at the onset of the symptoms were significantly lower in patients with severe and moderate disease than the patients with mild disease (p = 0.009, p < 0.001) (Table 1). There was a negative correlation between the SCORAD score and both age at admission (r = –0.277, p < 0.001) and age at onset of the symptoms (r = –0.474, p < 0.001) (Figure 1). Any significant relationship between the severity of disease and sex were not observed (Table 1).

**Table 1:** Comparison of the clinical and laboratory characteristics of patients in order to AD severity.

	Mild(n = 229)	Moderate & severe(n = 75)	p value
Age at admission (months)	7 (IQR: 5–11)	6 (IQR: 4-7)	0.009
Age at the onset of symptoms (months)	4 (IQR: 2.5–6)	2 (IQR: 1-3)	< 0.001
Sex of the patients (male/female) [n (%)/n(%)]	144 (62.9)/85 (37.1)	51 (68)/24 (32)	0.42
Patients with food allergy [n(%)]	53 (23.1)	68 (90.7)	< 0.001
Patients living in rural areas [n(%)]	56 (24.5)	37 (49.3)	< 0.001
Family history of atopy [n(%)]	40 (17.4)	50 (66.7)	< 0.001
Cigarette smoke exposure [n(%)]	130 (56.8)	68 (90.7)	< 0.001
Eosinophil count (μL)	170 (IQR: 80–405)	630 (IQR: 450–850)	< 0.001
Peripheral eosinophil percentage (%)	11 (IQR: 2–33.5)	49 (IQR: 31–65)	< 0.001

Abbreviations: Values in table are presented as the number of patients with/without the percentage in parenthesis, as the median with the interquartile range (IQR) in parenthesis as appropriate.

**Figure 2:**
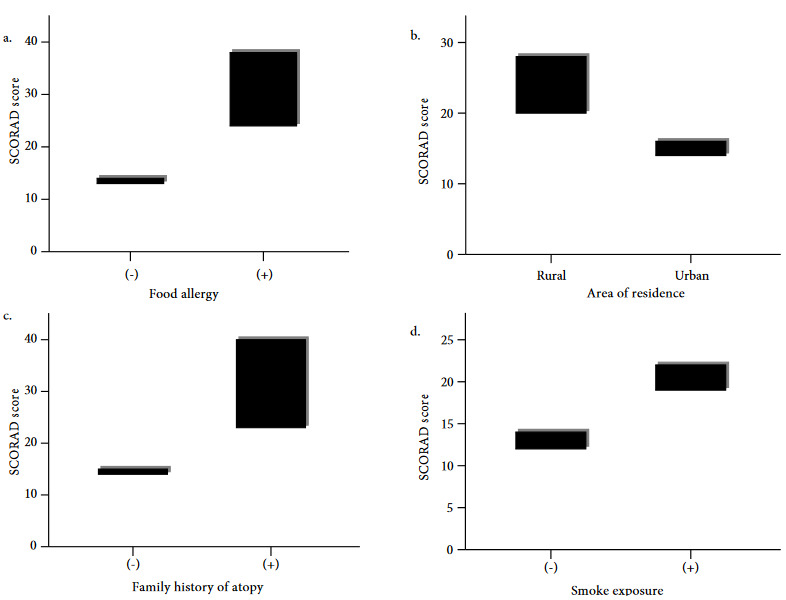
Comparison of SCORAD scores of patients in the context of (a) food allergy, (b) the area of residence, (c) family history of atopy, and (d) passive smoke exposure.

While 38% of the whole study group had FA, this rate was 23.1% in mild cases and 90.7% in moderate and severe cases (Table 1). Food sensitization rates were higher in individuals with moderate and severe disease (p < 0.001) and patients with FA had significantly higher SCORAD scores [33 (IQR: 22–44) vs 14 (IQR: 12–16); p < 0.001] (Figure 2a). Furthermore, the median age at the onset of AD symptoms was significantly lower for individuals with FA than the ones without FA [5 (IQR: 2–4) vs. 2 (IQR: 3–7); p < 0.001]. 

The majority of severely affected patients were living in rural areas (Table 1) and concerning the whole study group, it was observed that the ones living in rural areas had higher SCORAD scores than the individuals living in urban areas [22 (IQR: 15–39.5) vs 15(IQR: 12–22); p < 0.001] (Figure 2b). 

The frequency of familial atopy was 29.6% and significantly higher in the ones with moderate and severe disease than the mild disease (66.5% vs. 17.5%; p < 0.001) (Table 1). Moreover, the SCORAD scores of the patients with familial atopy history were notably higher (35.5 (IQR: 21.75–46) vs. 14 (IQR: 12–19); p < 0.001) (Figure 2c).

**Figure 3:**
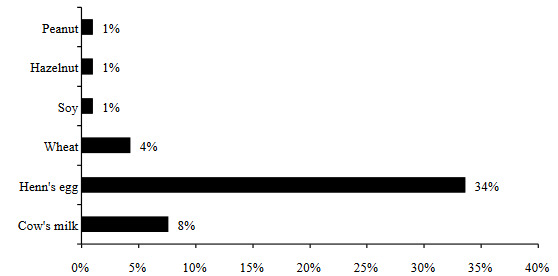
Distribution of the frequency of food sensitivity in patients according to the food type.

Sixty-five percent of the patients in the entire study group were exposed to passive cigarette smoking. The frequency of tobacco exposure was remarkably higher in severe and moderate patients than mild patients (90.7% vs. 56.8%; p < 0.001) (Table 1). Likewise, SCORAD scores were higher in patients exposed to passive cigarette smoking [21 (IQR: 14.75–38) vs. 13 (IQR: 12–16); p < 0.001] (Figure 2d). Egg and milk were the leading foods to which the patients were sensitive (Figure 3). 

**Figure 4:**
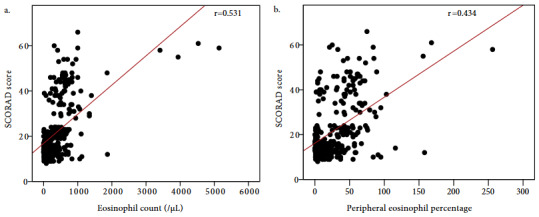
Correlation between SCORAD scores and (a) the eosinophil count, (b) peripheral eosinophil percentage (r, Spearman’s rank correlation).

The eosinophil count and peripheral eosinophil percentages were significantly higher in severe and moderate cases than mild ones [630 (IQR: 450–850)/μL vs. 170 (IQR: 80–405)/μL; p < 0.001 and 49 (IQR: 31–65)% vs. 11 (IQR: 2–33.5) %; p < 0.001] (Table 1). Both the eosinophil count and percentage found to be positively correlated with SCORAD scores (r = 0.531, p < 0.001 and r = 0.434, p < 0.001) (Figure 4).

In the multi-regression analysis, we observed that earlier onset of symptoms, having familial atopy, FA, and higher eosinophil counts were independently associated with SCORAD scores (Table 2).

**Table 2:** Linear regression analysis of factors associated with greater SCORAD scores.

	Univariate linear regression			Multiple linear regression		
	B coefficient	95% CI	p	B coefficient	95% CI	p
Age at the onset of symptoms	–0.396	–1.93 to 1.045	< 0.001	–0.74	–0.58 to 0.01	0.04
Food allergy	0.74	17.76 to 21.75	< 0.001	0.50	11.1 to 15.54	< 0.001
Sex	–0.04	–4.10 to 2.01	0.5			
Region of residency	–0.26	–10.93 to 4.39	< 0.001	–0.05	–3.40 to 0.49	0.14
Family history of atopy	0.54	7.28 to 10.41	< 0.001	0.20	3.42 to 7.98	< 0.001
Cigarette smoke exposure	0.38	7.62 to 13.3	< 0.001	0.04	–0.80 to 3.20	0.23
Eosinophil count	0.54	0.011 to 0.015	< 0.001	0.27	0.005 to 0.008	< 0.001

Abbreviations: SCORAD, scoring atopic dermatitis ındex, CI: coefficient interval.

## 4. Discussion

In the present study, we assessed the factors associated with the severity of AD in children. Patients with earlier onset of the symptoms, food sensitivity, positive familial history for atopy, rural area of residence, cigarette smokes exposure, higher blood eosinophil levels were found to be more prone to have severe AD.

We analyzed the severity of AD in our patients by SCORAD index, which has been reported as one of the most reliable and practicable methods [8,11]. One fourth of the participants in this study were moderate-severe cases. This rate is lower than what is reported in the former study by Ricci et al. [12], which was performed in a pediatric population with a similar number of cases. The previous studies conducted on pediatric AD patients in Turkey have also reported larger proportions of severe cases [13–15]. In the UK, however, the rates of mild, moderate, and severe AD in children reported to be 82%, 12%, and 6%, respectively; which are comparable to our results [16]. Hence, these differences could be attributed to variations in methodology and populations.

The ratio of mild to severe AD patients likely increases with age [16]. In our study, the individuals showing symptoms at an earlier age had higher SCORAD scores, and the median age of the severe-moderate cases was found to be lower than the mild cases. The regression analysis revealed that younger age was an independent factor associated with a more severe clinical picture in AD. Therefore, cases with early onset of AD symptoms should be monitored more closely due to a high possibility of a severe and persistent clinical course. 

Although some studies report slightly female predominance [17,18], the majority of the patients, particularly those with severe disease were male in our study population. Besides, in consistency with the former studies [19], we couldn’t reveal any significant relations between the severity of AD and patient’s sex.

FA can coexist with AD in infancy [20]. In two-fifths of our patients, AD is accompanied by food sensitivity, and consistent with the literature [16], egg and milk sensitivity were the most common triggers prevalent. Almost all of our moderate and severe cases had FA. These results were similar to previous studies, suggesting that in the first 2 years of life, the majority of infants with moderate to severe disease show sensitization to food allergens, whereas those with mild disease are less sensitized [21,22]. In a retrospective study, it was observed that the patients with FA developed AD at earlier ages [23]. Similar to this study, we revealed that AD symptoms appeared earlier in the cases accompanied by FA, and severity scores of these cases were significantly higher than the ones without FA.

Recently the prevalence and incidence of AD have been increasing. A major theory explaining this trend, in general, is the ‘hygiene hypothesis’ that supports an inverse relationship between AD and exposure to endotoxin, early daycare, farm animal, and dog pets in early life [24,25]. As part of the ‘hygiene hypothesis,’ it has been thought that AD is more common in urban than in rural communities [26]. In the context of this hypothesis, we analyzed the association between the area of residence and the severity of AD. Contrary to the previous studies, we found that individuals living in rural areas had higher SCORAD scores than those living in urban areas. Longitudinal cohort studies conducted in this particular region of Turkey are needed to explain this difference and reveal any causation.

Population-based studies have indicated that the strongest risk factor is a positive family history for atopic diseases, particularly for AD [27]. Approximately 70% of individuals with AD have a positive family history of atopic diseases [28]. Patients with one atopic parent have two to threefold increased risk of developing AD. Moreover the risk increases to three to fivefold in case both parents are atopic [1]. In our study, almost all of the severe cases had a positive family history of atopic diseases. The SCORAD scores of the patients with familial atopy history were significantly higher. We can postulate that positive family history for atopic diseases increases the possibility of a more severe clinical course for patients with AD.

Early studies have shown that cigarette smoke in the environment increases children’s risk of allergic sensitization [29]. A meta-analysis reviewed 86 studies, from 39 countries, investigated the association of active smoking, passive smoking exposure, and maternal smoking during pregnancy with childhood AD, and concluded that childhood AD was significantly associated with passive smoking exposure [30]. In our study, 90.7% of severe and moderate cases had passive cigarette smoking exposure and those with passive cigarette smoking exposure had a significantly higher SCORAD score. These results indicate that passive tobacco exposure increases the severity of AD. Therefore, it is strongly recommended that all caregivers should stop smoking.

Eosinophilia in patients with AD is usually mentioned as a nonspecific finding [31]. However, recent studies have determined a positive correlation between peripheral eosinophilia and the severity of AD [13,32]. Comparable to these studies, we found that the blood eosinophil count and the eosinophil percentage of moderate and severe cases were higher than mild cases. Besides, a positive correlation was established between patients’ SCORAD scores and the eosinophil counts and percentages. Since they can be practically evaluated and have potential utility in clinical practice; blood eosinophil count and percentage can be very valuable parameters for assessing the severity of AD in children.

As far as we know, this study was one of the few studies evaluating children with AD in this particular region of Turkey. Because of the retrospective cross-sectional nature and relatively small sample size of our study, our findings on investigating possible factors associated with the increased severity of AD were limited. Especially, the socioeconomic or cultural level of the patients, that may affect the parameters (such as passive cigarette smoking, living in rural areas) that we consider in assessing the severity of AD could not be evaluated within the scope of our study. Further studies are still needed to clarify the factors playing a role in severe AD in the pediatric population. 

## 5. Conclusion

Our findings show that early-onset, food sensitivity, having familial atopy history, and passive smoke exposure plays a significant role in severe AD, consistent with previous reports. On the contrary, living in rural areas was found to be associated with severe AD. Since it is remarkably correlated with SCORAD scores, we can postulate that eosinophil count can be used as a marker to assess the severity of AD in children.

## Informed consent

Informed consent was obtained from all caregivers of the participants included in the study. This retrospective study was approved by local ethics committee at Artuklu University with the decision number 2020/9-4.
